# Non-Proteasomal UbL-UbA Family of Proteins in Neurodegeneration

**DOI:** 10.3390/ijms20081893

**Published:** 2019-04-17

**Authors:** Salinee Jantrapirom, Luca Lo Piccolo, Masamitsu Yamaguchi

**Affiliations:** 1 Department of Pharmacology, Faculty of Medicine, Chiang Mai University, Chiang Mai 50200, Thailand; 2 Department of Neurotherapeutics, Osaka University Graduate School of Medicine, Osaka 565-0871, Japan; lopiccolo@neurother.med.osaka-u.ac.jp; 3 Department of Applied Biology, Kyoto Institute of Technology, Kyoto 606-8585, Japan; myamaguc@kit.ac.jp

**Keywords:** UbL-UbA, non-proteasomal ubiquitin receptor, proteostasis, ubiquitin-like, neurodegenerative diseases, *Drosophila*

## Abstract

Ubiquitin-like/ubiquitin-associated proteins (UbL-UbA) are a well-studied family of non-proteasomal ubiquitin receptors that are evolutionarily conserved across species. Members of this non-homogenous family facilitate and support proteasomal activity by promoting different effects on proteostasis but exhibit diverse extra-proteasomal activities. Dysfunctional UbL-UbA proteins render cells, particularly neurons, more susceptible to stressors or aging and may cause earlier neurodegeneration. In this review, we summarized the properties and functions of UbL-UbA family members identified to date, with an emphasis on new findings obtained using *Drosophila* models showing a direct or indirect role in some neurodegenerative diseases.

## 1. Introduction

Protein homeostasis (proteostasis) is a fine-tuned process that controls the biogenesis, folding, trafficking and degradation of proteins [1,2]. Proteasomal degradation is one of the mechanisms by which cells regulate the abundance of proteins and/or eliminate those that are no longer functional [3].

The proteasome is a large multi-subunit complex that selectively degrades approximately 80–90% of misfolded or damaged cellular proteins in a ubiquitin (Ub)- and ATP-dependent manner and also provides proper Ub recycling [1,4,5,6,7]. The 26S proteasome is a major proteasome consisting of a 20S proteasome core particle with peptidase activity and two 19S-regulating particles [6,8,9]. The intracellular elimination of toxic protein aggregates may also be performed by autophagy, a process by which superfluous or potentially dangerous cytoplasmic materials are delivered to lysosomes for degradation [10].

The ubiquitin proteasome system (UPS) needs ubiquitylation to covalently mark substrates with Ub before degradation [11]. The Lys48-linked Ub chain with a minimum length of four tagged Ub appears to be a predominant signal for protein degradation; however, recent studies have shown that other Ub-linked chains facilitate degradation by the 26S proteasome [12,13]. Canonically, ubiquitylated proteins are digested by the 26S proteasome to short peptides, 90% of which range between two and ten residues in length [14]. In a few cases, the 26S proteasome drives incomplete degradation by a process referred to proteasomal processing that eventually releases protein fragments with new cellular functions, such as the p105 and p100 precursors of the p50 and p52 subunits of NFkB, which function in the immune system, and Gli2 and Gli3, which function in hedgehog signaling [15,16,17,18,19,20]. Recent studies using in vitro models have been clarifying the processivity of the proteasome to elucidate whether low complexity domain-containing substrates (or prion-like domains, PrLD) undergo incomplete proteasomal degradation [21]. The mechanisms underlying the proteasomal partial degradation of so-called “slippery substrates” remain elusive and need further investigation; however, this is now emerging as an important aspect in this research field, with the accumulation of PrLD-proteins being a feature of several neurodegenerative diseases.

UPS mainly targets soluble and monomeric proteins over aggregated ones by a mechanism involving heat shock 70-kDa protein (HSP70) and the sequential actions of three classes of ubiquitin ligases: E1, E2, and E3. Degradation by UPS requires ubiquitin-activating enzyme (E1), which binds and activates Ub in an ATP-dependent mechanism. Ub is then transferred to an active site of ubiquitin-conjugating enzyme (E2), and ubiquitin protein ligase (E3) ultimately transfers Ub directly or indirectly from E2 to protein substrates [11].

Specific subunits of 19S-regulating particles have been identified as Ub receptors with the ability to recognize free Ub and Ub-conjugated proteins. Rpn10 and Rpn13 are the most well-studied proteasomal Ub receptors and appear to be evolutionarily conserved across species from yeast to mammals [22,23,24]. Non-proteasomal Ub receptors also exist and play a critical role in supporting or facilitating proteasomal degradation. The most well-characterized of these proteins is the Ubiquitin-like (UbL)-Ubiquitin associated (UbA) family. This family of proteins are not only involved in proteasomal degradation, but also play roles in a number of additional cellular processes, such as nucleotide excision repair (NER), spindle pole body duplication, and cell growth, which cumulatively make this family a very versatile group of proteins. At least 5 major members belonging to the non-proteasomal UbL-UbA family have been identified to date, with Rad23 and Ubiquilin2 being the most well-characterized.

**Figure 1:**
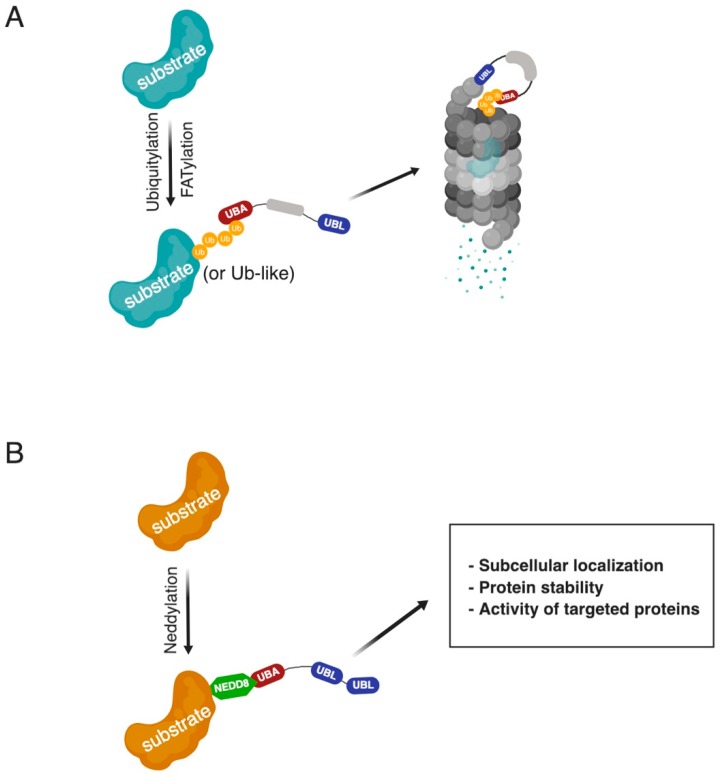
Paradigm towards the regulation of UbL-UbA proteins. (**A**) Ubiquitylated and FATylated substrates are bound to UbL-UbA proteins before their degradation by proteasomes. Like the ubiquitylation, “FATylation” is a post-translational modification in which FAT10 forms a covalent bond with its target proteins. (**B**) Neddylated substrates are bound to the UbA domain of NUB1 in order to facilitate localization or stability and promote other activities.

The UbA domain binds mono- and poly-ubiquitin chains as well as ubiquitylated proteins, while the UbL domain interacts with the ubiquitin-interacting motif (UIM) domain of the proteasomal Ub receptor Rpn10. UbA domains contain a special structure of hydrophobic patches that provide stronger binding to Lys48-linked polyubiquitin chains than to mono-Ub [25,26,27] (Figure 1A). The UbL domain primary structure ranges from 45 to 80 amino acids with very high similarity to the Ub sequence; thus, both may compete for binding to protein targets [28].

**Figure 2:**
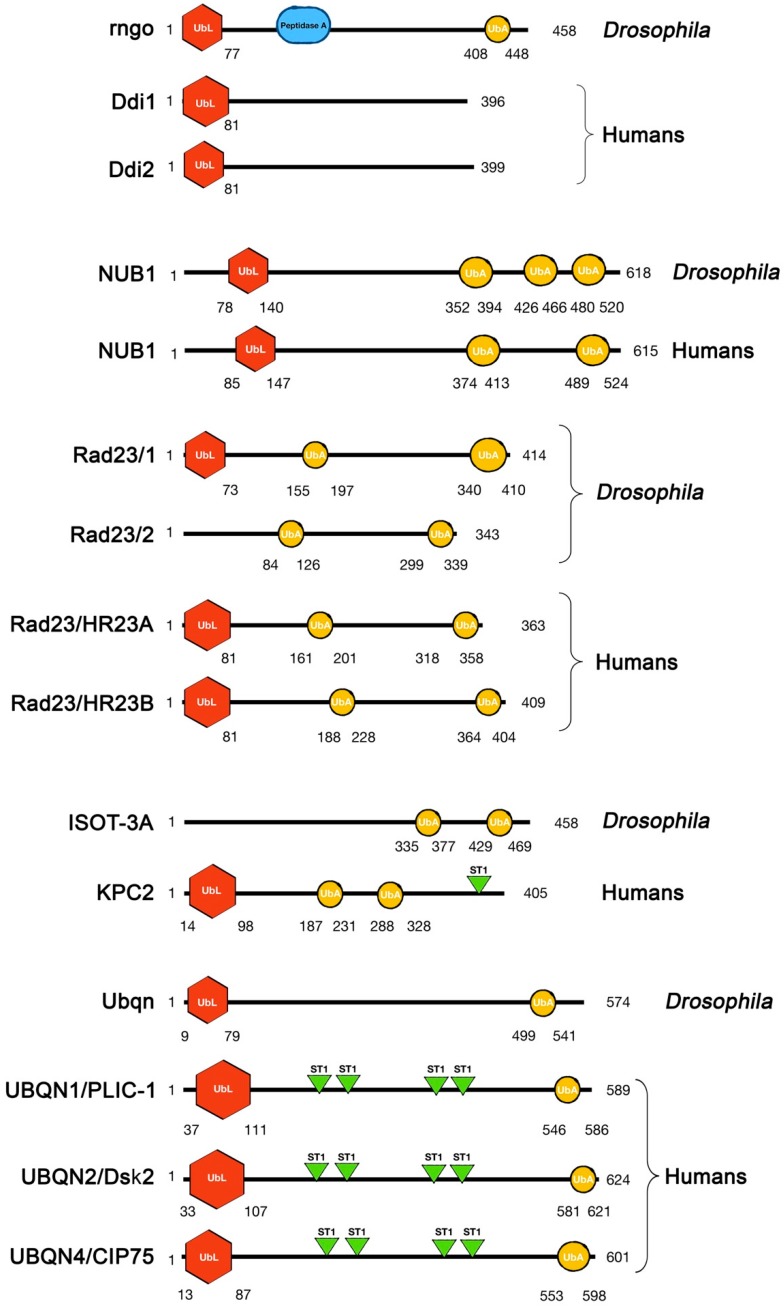
Comparison of *Drosophila* and human UbL-UbA proteins.

Any attempts to catalog UbL-UbA-bearing proteins in a single group may be far from useful or precise. Beyond structural analogies and sequence similarities in their domains (Figure 2 and Table 1), these proteins have different roles and targets and also contribute to proteasome activity in different manners from each other. For example, some may facilitate or inhibit proteasomal function depending on the substrates. The role of UbL-UbA remains complex and an area of active debate under continued revision.

The primary structures of UbL-UbA members were analyzed by Expasy Prosite (www.expasy.org) to search for the presence and localization of relative domains.

Due to the broad physiological implications of protein homeostasis pathways, dysregulation of proteostasis is often involved in the development of multiple pathological conditions, including neurodegenerative diseases, such as Alzheimer’s disease (AD), Parkinson’s disease (PD), frontotemporal dementia (FTD), Huntington’s disease (HD), Amyotrophic Lateral Sclerosis (ALS), and prion diseases, which are collectively classified as proteinopathies [29,30,31,32]. Proteinopathies share a common pathological hallmark that comprises poly-ubiquitylated misfolded protein aggregation. Therefore, the most common protein associated with aggregates is ubiquitin, indicating a role for UPS in proteinopathy-related pathogenesis. Emerging evidence revealed a pivotal role for conserved Ubiquilin2 in ubiquitin pathologies associated with aggregates of the RNA-binding protein TDP-43, with the loss of Ubiquilin2 being the cause of a gain of neurotoxic TDP-43 function [33,34]. Similar studies conducted to gain insights into the mechanisms of other UbL-UbA proteins may reveal the important contribution of this family’s proteins to neurodegenerative diseases.

In this review, we summarized the properties and functions of different UbL-UbA members, with an emphasis on new findings obtained using *Drosophila* models showing direct or indirect roles in neurodegenerative diseases.

## 2. Physiological Roles of UbL-UbA Family Proteins and Their Contribution to Diseases

### 2.1. Ddi

The DNA damage-inducible protein (Ddi1) was initially discovered and mostly studied in yeast [35,36,37]. There are two Ddi isoforms in humans, referred to as Ddi1 and Ddi2, with the first being the more extensively studied. Ddi1 is normally found in the nucleus, but some of its functions are linked to various proteins in the cytoplasm [38]. In silico analyses are not capable of outputting a classic UbA domain in the C-terminal region of Ddi isoforms due to low sequence similarity (Figure 2). However, previous studies reported that the weakly conserved UbA domain of Ddi1 formed a characteristic UbA:Ubiquitin complex [39,40] by binding K48-linked polyubiquitin chains [41]. In addition, despite having a ubiquitin-like fold, the UbL domain of Ddi isoforms did not interact with typical proteasomal Ub receptors, but exhibited the ability to bind ubiquitin [39]. More recent structural studies on the enigmatic Ddi protein have identified a novel ubiquitin-interacting motif (UIM) located at its C-terminal region with a weak yet specific affinity towards ubiquitin [42]. Moreover, the central portion of the primary structure of Ddi1 contains a retroviral protease-like (RVP) domain that is required for protein homodimerization to facilitate some cellular functions related to the cell cycle [43]. Its possession of the RVP domain indicates a proteolytic function for Ddi1 during protein turnover [44].

Few Ddi substrates have been studied to date in mammals. A weak relationship was previously reported with the proteasomal regulatory protein Rpn1 [45,46], and the function of Ddi in proteasome activity or the ubiquitin pathway needs to be more fully characterized. Recent studies on *Caenorhabditis elegans (C. elegans)* revealed that Ddi1 indirectly involves in proteasomal function by activating SKN1, a transcription factor related to mammalian Nrf1/2 [47]. During proteasomal impairment, the Ddi1 of *C. elegans* exhibits the ability to cleave the proteasome activator SKN1 via its aspartic protease activity and activates an endoplasmic reticulum (ER)-associated isoform of SKN1 [47]. Mammalian Nrf1 is also associated with ER and is subjected to proteolytic cleavage [48,49,50], suggesting a conserved mechanism of proteasome surveillance that warrants future investigations to clarify the role of mammalian Ddi1 in similar mechanisms.

Ddi1 appears to contribute to neurodegenerative diseases. For example, in a specific familial variant with the neuroradiological features of AD, but lacking amyloid-β deposits in the brain, whole-exome sequencing revealed a novel nonsense mutation in the *Ddi1* gene of patients conferring a gain of Ddi function that may play role in this kind of dementia [51].

*Drosophila* carries one Ddi1 homolog that was identified in 2011 as CG4420 and molecularly characterized as a ubiquitin receptor with the ability to bind to Rpn10 [52]. CG4420 was successively referred to as Rings lost (Rngo) because its mutation causes the loss of a germline cell’s connection [52].

By employing an unbiased ubiquitin proteomic approach, further studies demonstrated that Rngo was a target of the E3 ligase UBE3A [53] and, interestingly, UBE3A-mediated Rngo ubiquitylation was shown to be conserved in SH-SY5Y neuroblastoma cells in which the human UBE3A homolog also has the ability to target and ubiquitylate Ddi1 [53]. These findings are very important in the neurological field because mutations in UBE3A are associated with Angelman syndrome, a complex neurodevelopmental disorder [54], and the newly identified UBE3A target Ddi1 was also shown to be temporally regulated during neuronal development. Emerging evidence is suggesting that Ddi1 has biological functions not yet described that may be of relevance for clinical research on Angelman syndrome. The use of new *Drosophila* models of Rngo may expand our understanding of the functions of Ddi1 in neurological diseases.

### 2.2. NUB1

Nedd8 Ultimate Buster 1 (NUB1) is a predominately nuclear-located protein that is mainly involved in the regulation of ubiquitin-like proteins, such as Neural Precursor Cell Expressed Developmentally Down-regulated 8 (NEDD8) and FAT10, with high levels of NUB1 accelerating the degradation of NEDD8 (Figure 1B), FAT10, and their conjugated targets in a proteasomal-dependent manner [55,56,57,58,59,60].

The N-terminal UbL domain of NUB1 may bind Rpn1 and Rpn10 proteasomal subunits, while the C terminus carries two UbA domains that are involved in the interaction with ubiquitin-like proteins, as described above. Recent findings demonstrated that the neddylation activity of NUB1 is under the control of the oncogenic E3 ubiquitin ligase Mdm2. Mdm2 acts as a positive regulator of NUB1 function because di-ubiquitylation on NUB1 lysine-159 by Mdm2 leads NUB1 to negatively regulate Nedd8 and neddylated target proteins [61].

A longer unsliced variant of NUB1 (NUB1L) has also been identified and characterized [62]. NUB1L down-regulates the protein levels of NEDD8 and neddylation by specifically recognizing NEDD8 and the valosin-containing protein (VCP/p97). In coordination with the VCP/p97-UFD1-NPL4 complex, NUB1L has the ability to promote the transfer of NEDD8 to proteasomes for degradation [63]. VCP/p97 is a chaperone protein that regulates ubiquitin-dependent protein degradation [64,65], highlighting an important role for VCP/p97 in the ubiquitylation and neddylation pathways. Collectively, these findings confirmed that NUB1L promotes the degradation of misfolded proteins [66].

The role of NUB1 in the regulation of neddylated substrates remains an area of active investigation. The biological outcomes of post-translational modifications such as ubiquitylation and neddylation appear to be diverse, from protein destruction to transcriptional regulation, subcellular localization, DNA repair, endocytosis, signal transduction, and autophagy. Neddylation of the tumor suppressor p53 is mediated by NUB1 in order to regulate p53 localization and inhibits its transcriptional activity rather than promote its degradation via proteasomes [67] (Figure 1B).

The involvement of NUB1 in neurological diseases associated with the formation of toxic aggregates has been intensively investigated. The immunohistochemical characterization of eosinophilic intranuclear inclusions (INI) in the brains of patients with intranuclear inclusion body disease (INIBD) revealed high levels of NUB1 and NEDD8, suggesting a role for neddylation in the formation of this class of protein aggregate [68]. In patients with PD and dementia with Lewy bodies (DLB), NUB1 has been found to co-localize with inclusions [69] and accumulates in a the presynapses of the hippocampus, cerebral neocortex, and substantia nigra, at which some toxic species of the proteinase K-resistant α-synuclein are deposited [70]. NUB1 may suppress the formation of Synphilin-1-positive inclusions. Since Synphilin-1 is a major component of inclusion bodies in the brains of patients with neurodegenerative α-synucleinopathies, including PD, the role of NUB1 in these neurodegenerative diseases may be relevant. Biochemical assays revealed that NUB1 targets Synphilin-1 to proteasomes for its efficient degradation, decreases in Synphilin-1 levels and suppresses the formation of Synphilin-1-positive inclusions [71].

The role of NUB1 in the toxic aggregation of the microtubule-associated protein Tau has been also investigated. The abnormal phosphorylation and aggregation of Tau have been associated with neurodegenerative diseases, including AD and frontotemporal lobar degeneration (FTLD). Among the numerous kinases that phosphorylate Tau such as glycogen synthase kinase 3β (GSK3β) is strongly expressed in the brain and is involved in the hyperphosphorylation of Tau, with an increase in GSK3β levels being observed in AD [72,73,74,75].

NUB1 abolishes the recruitment of GSK3β to Tau inclusions by disrupting the Tau-GSK3β interaction. Moreover, NUB1 lowers the GSK3β-mediated phosphorylation of Tau, leading to a reduction in Tau levels in intracellular inclusions [76]. Therefore, NUB1 is a key player in neurodegeneration associated with the Tau pathology. Since GSK3β is an important effector of numerous signaling pathways, it is of interest to clarify whether NUB1 affects other GSK3β-related pathways.

The *CG15445* gene is the *Drosophila* homolog of *NUB1*, which has been referred to as *Drosophila NUB1* following the study by Lu and colleagues in which a *Drosophila* model of HD was employed to identify gene modifiers of the toxicity associated with high levels of the mutant form of the protein huntingtin (mHTT) [77]. Huntington’s disease (HD) is caused by a CAG trinucleotide repeat expansion in the *HTT* gene that leads to the formation and neurotoxic accumulation of polyglutamine-expanded mHTT [78]. Genome-wide RNA interference screening with a well-characterized *Drosophila* model of HD allowed Lu and colleagues to confirm that NUB1 reduced mHTT levels [77]. Consistent with the function of NUB1 in promoting the degradation of neddylated proteins, NUB1 facilitates HTT clearance by recruiting the neddylated ubiquitin E3 ligase CUL3 (Nedd8-CUL3) to HTT and enhancing ubiquitylation, thus the clearance of HTT [77]. NUB1 also mediates the targeting of mHTT to the activated NEDD8-conjugated CUL3 E3 ubiquitin ligase complex. Therefore, NUB1 promotes the proteasomal degradation of polyubiquitylated mHTT [77].

NUB1 was previously reported to be inducible by interferon-β (IFNβ) [55], and rodent models of HD showed that an IFNβ treatment promoted NUB1-mediated reductions in mHTT [77]. Therefore, the targeting of NUB1 may be regarded as a promising therapeutic strategy applicable to a broad range of diseases associated with the expression and accumulation of toxic proteins that will be degraded.

### 2.3. Rad23

Mammalian cells express two homologs of yeast Rad23, the so-called “homolog of Rad23” (HR23) proteins. The *HR23* gene, hereafter called *Rad23* is evolutionarily conserved among eukaryotic organisms. Rad23 A and B, both are essential for DNA repair and protein degradation [79,80].

The first indication that Rad23 is involved in proteolytic degradation via the proteasome was provided by the interaction between its UbL domain and the ubiquitin-binding site of S5a, a human homolog of yeast Rpn10 [81]. Since then, the link between Rad23 and proteolytic degradation has been widely investigated by several methods, such as yeast two-hybrid screening and co-affinity purification.

The C-terminal portion of Rad23 contains two UbA domains [40] (Figure 2), which are required for binding with ubiquitylated proteins and lead to the degradation or stabilization of its protein targets. Previous studies demonstrated that Rad23 bound some proteasomal components to translocate ubiquitylated proteolytic substrates to the proteasome through a combination between UbL–proteasome and UbA–(poly)-ubiquitin binding [82,83,84]. Through its N-terminal UbL domain, Rad23 interacts with Rpn1, one of the regulatory subunits of the proteasome [85], while the UbA domain is involved in binding with mono- and poly-ubiquitin [40,82,86,87,88], with the Lys48-linked multi-ubiquitin being the preferred substrate [89,90].

The UbL domain of Rad23 also exhibits some regulatory activity because the phosphorylation of its serine residues may inhibit the interaction of Rad23 with the 26S proteasome [91], while its overexpression impairs substrate delivery to the proteasome [82]. The UbA domain of Rad23 functions as a cis-acting stabilization signal to protect itself from proteasomal degradation [90].

The role of Rad23 in DNA repair has been extensively documented. The discrimination of the appropriate excision repair pathway according to the type of DNA lesion has proven to be the most important role for Rad23 [79].

Rad23 has been found to be one of delivery factors for ubiquitylated ER substrates after extraction by Cdc48-Ufd1-Npl4 complex in which Cdc48 is a homologue of VCP in yeast [92]. Rad23 works together with Cdc48 to ensure that their substrates can be reach an efficient degradation [93]. Moreover, the efficient of ER-related glycosylated rich A chain degradation also requires facilitation between Rad23 and Png1, a deglycosylating enzyme [94,95]. The overexpression of Rad23 results in a marked increase in ubiquitylated proteins, with an even higher level being observed in the UbL deletion mutant [96]. However, the loss of Rad23 also results in the accumulation of poly-ubiquitylated proteins, such that an imbalance in Rad23 may affect proteasomal functions depending on its substrates and interactors. For example, through the UbA domain, Rad23 binds and prevents the de-ubiquitylation of poly-ubiquitylated p53. However, while previous studies reported that the depletion of Rad23 reduced the level of p53 [97], others showed that the overexpression of Rad23 also resulted in its accumulation [98].

Previous studies demonstrated that the proteasomal degradation of ataxin-3 is regulated by a direct interaction between its UbS2 domain and Rad23 [99]. Since the expansion of the CAG tract in the *ataxin-3* gene (*ATXN3*) leads to the translation of a pathogenic form with expanded polyQ referred to as SCA3 [100], Rad23 activity has proven to be critical for modulating SCA3 turnover in *ATXN3* expansion mutations.

The *Rad23* gene was identified in *Drosophila* in 1999 by Nabirochkina EN et al. It was characterized as a gene involved in proteasomal-related functions due to its up-regulation caused reduction in proteasomal activity [101]. *Drosophila* carries three Rad23 isoforms referred to as Rad23/1, 2, and 3, respectively (Figure 2), which are similar to the human isoforms. The UbL and UbA domains of Rad23/1 are similar to those of isoform A of human Rad23 (Table 1).

Regarding the SCA3 pathology, *Drosophila* models have been employed to identify Rad23 as the molecular partner of ataxin-3 [102] and investigate whether the Rad23 interaction with SCA3 is critical for ataxin-3-dependent toxicity [103]. The modulation of Rad23 levels has been shown to influence the toxicity of ataxin-3 in *Drosophila*, such that reductions in Rad23 levels alleviated toxicity in this SCA3 model [103].

### 2.4. KPC2

UbA domain-containing 1 (UBAC1) contains one NH_2_-terminal UbL, two UbA, and one COOH-terminal STI1 (heat shock chaperonin-binding) domains (Figure 2). Due to its ability to bind to and stabilize the protein KPC1 in order to form the Kip1 ubiquitylation-promoting complex (KPC), UBAC1 is also known as KPC2 [104,105]. KPC regulates the degradation of the cyclin-dependent kinase inhibitor p27 at the G_1_ phase of the cell cycle and, thus, plays a role in controlling cell proliferation. KPC2 has been shown to promote the transfer of p27 molecules that have been ubiquitylated by KPC1 to the 26S proteasome [106]. Therefore, the overexpression of KPC promoted p27 degradation, whereas the dominant-negative mutant and RNAi of KPC inhibited p27 degradation [104].

More recently, in a proteome-wide interactome approach, KPC was shown to play a role in the regulation of HOXA2, a transcriptional factor belonging to the HOX family of proteins, which are fundamental for development [107]. In contrast to its role in p27 degradation, KPC2 exerted two main effects on HOXA2; reductions in its transcriptional activity and the induction of a HOXA2 nuclear exit [107]. Despite the limited characterization of KPC2 interactors and ubiquitylated targets, the cellular function of KPC2 appears to be very important for controlling cell cycle progression and development.

The homologue of KPC2 in *Drosophila* is the isopeptidase-T-3 (ISOT-3A) protein, which contains two UbA domains on the C terminus, but lacks the UbL domain (Figure 2). However, ISOT-3A has not yet been investigated.

### 2.5. Ubiquilin (UBQLN)

Mammals carry five *UBQLN* genes, of which *UBQLN1*, *UBQLN2*, and *UBQLN4* are widely expressed in various tissues of human, mice, and rats, while *UBQLN3* is exclusively expressed in the testes of both human and mice [108,109,110], whereas *UBQLNL* is still under-investigation to date.

UBQLN2 or Ubiquilin2 (sometimes referred to as Dsk2, PLIC-2, or CHAP1) is the most well-characterized of the five ubiquilins and perhaps the most well-studied UbL-UbA protein because mutations in the *UBQLN2* gene have been identified in rare cases of X-linked juvenile and adult forms of ALS, ALS/FTD [111], and the atypical hereditary spastic paraplegia phenotype [112]. Moreover, UBQLN2 shows a propensity for self-assembly and aggregation in neurodegenerative diseases [113].

UBQLN2 regulates the protein degradation of ubiquitylated targets not only by proteasomes [114,115,116,117], but also through other pathways, including endoplasmic reticulum-associated protein degradation (ERAD) and macroautophagy [118,119,120,121,122]. Certain targets of UBQLN2 require UBQLN2 to join the HSP70-HSP110 disaggregase machinery in order to be degraded by proteasomes [114]. A recent study reported that UBQLN4 attenuated p21 proteasomal degradation by interacting with the E3 ubiquitin ligase RNF114, indicating a critical role for UBQLNs in the regulation of the cell cycle and cellular senescence [123].

In addition to their function in controlling protein turnover and homeostasis, UBQLNs have several other distinct activities that are important in various cellular pathways. UBQLNs associate with actin and intermediate filaments [119] and localize inside aggresomes [120]. Moreover, UBQLN1 in combination with protein-disulfide isomerase (PDI) plays an important role in the regulation of cell death [120,121]. A novel function for UBQLN has recently been reported to drive gene transcription when complexed with the dHP1c isoform and the transcription factors, WOC and Relative-of-WOC (ROW) [124].

The UbL domain of UBQLN2 binds to several proteasomal subunits and E3 ligase. It also interacts with the ubiquitin-interacting motif (UIM) of several endocytic proteins such as Eps15, Hrs, and STAT2/Hrp [116,125,126,127]. The UbA domain of UBQLN2 interacts with presenilins [108,128] and the GABA-A receptor [129]. It also binds to mono- and polyubiquitin chains and provides an additional binding site for proteasomal subunits [115,130].

The large STI-like chaperonin-binding region in the central portion of UBQLN2 interacts with the HSP70-like chaperone Stch and is important for ubiquilin multimerization and facilitation of the ERAD pathway [119,131]. The collagen-like triple helix region, which is found exclusively in UBQLN2, has been implicated in familial cases of ALS/FTD [111].

Several lines of evidence have linked ubiquilins to diverse neurodegenerative diseases, such as AD [132,133] as well as ALS and FTD [134,135]. For example, the overexpression of *UBQLN1* or *UBQLN2* leads to the accumulation of AD causative presenilin proteins by slowing down their proteasomal degradation [108,128,136,137]. As described above, missense mutations in the proline residues of PXX repeats or outside those repeats in UBQLN2 were found to cause sex-linked, dominant ALS, often associated with FTD [111,138,139,140]. Ubiquilin-containing aggregates have also been detected in ALS patients with hexanucleotide expansions in the non-coding region of the *C9orf72* gene, which is a commonly found mutation in both familial and sporadic ALS [141]. A loss-of-function and/or haploinsufficiency have been suggested as the main disease mechanisms for *UBQLN2* mutations in ALS patients [34]. Moreover, ubiquilins are sequestered into aggregates by proteins involved in spinocerebellar ataxia type 1 (SCA1) [142,143] and HD [144]. Recent findings confirmed that UBQLN2 is a polyubiquitin-TDP-43 co-chaperone with the ability to mediate the autophagosomal delivery and/or proteasome targeting of TDP-43 aggregates [145,146,147]. TDP-43 UBQLN2-positive inclusions with or without *UBQLN2* and *TDP-43* mutations have also been consistently identified in ALS and FTD patients, with high levels of the UBQLN2 wild type or mutant enhancing the cytosolic accumulation of TDP-43 [148,149,150].

**Figure 3:**
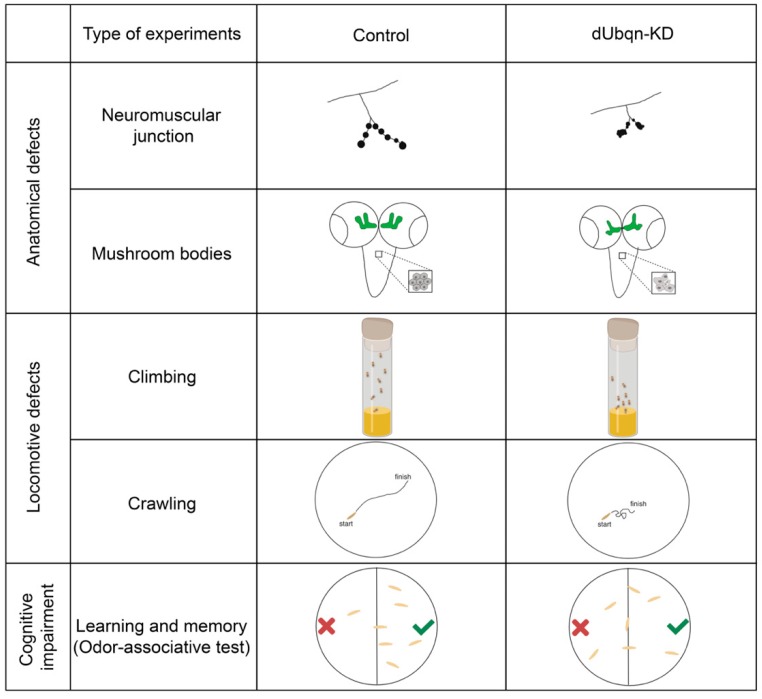
Overview of defects caused by UBQLN2 loss-of-function in *Drosophila* models [33,152].

*Drosophila* contains a single *UBQLN* gene homologue located on the X chromosome that is referred to as *dUbqn* and encodes a predicted protein of 547 amino acids [151], which shows high similarity to mammalian ubiquilins 1 and 2 (Figure 2, Table 1). The knockdown of *dUbqn* induces various changes in synaptic morphology, resulting in defects in locomotion and learning ability [152] (Figure 3).

**Figure 4:**
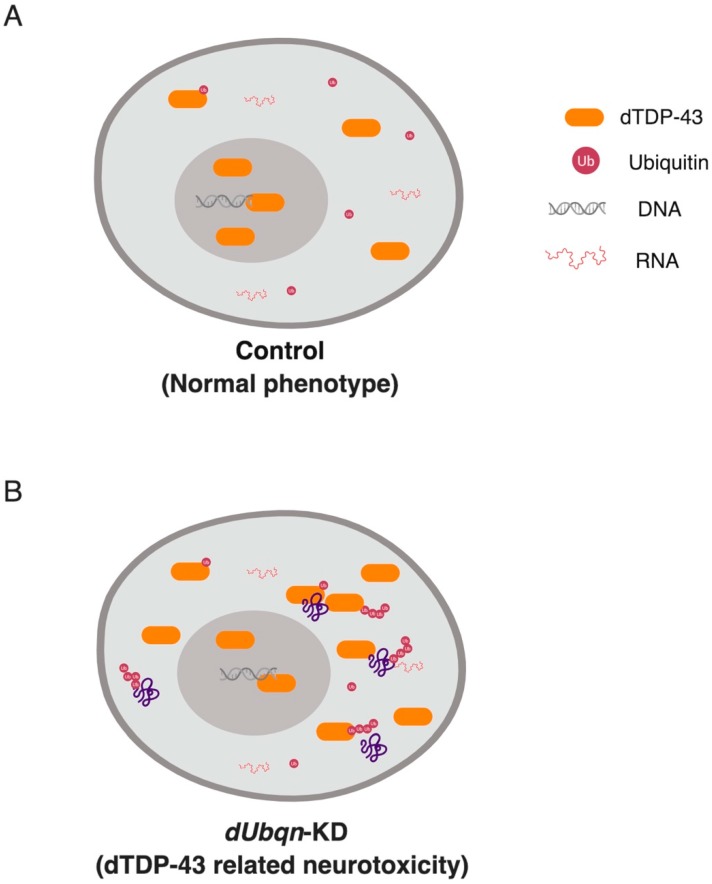
UBQLN2 loss-of-function causes a TDP-43-like pathology in the fly [33]. (**A**) Under physiological conditions, TDP-43 shuttles between nuclear and cytoplasmic compartments. (**B**) The depletion of *Drosophila* UBQLN2 functions by RNAi triggers the abnormal accumulation of toxic cytoplasmic TDP-43.

*Drosophila* models have been established to understand the contribution of UBQLNs to the proteostasis of TDP-43 and its toxicity in vivo (Figure 4). The co-expression of human UBQLN1 reduces steady-state levels of TDP-43, but, unexpectedly, increases the severity of TDP-43-induced phenotypes [146]. More recently, the depletion of dUbqn was shown to markedly affect the expression and sub-cellular localization of *Drosophila* TDP-43 (dTDP-43), resulting in a cytoplasmic ubiquitin-positive (Ub^+^) TDP-43 pathology [33]. Despite decreasing dUbqn levels markedly impairing the proteasome to promote the accumulation of diverse polyubiquitylated proteins, recent findings have shown that neurons lacking ubiquilin functions are particularly sensitive to the formation of cytoplasmic polyubiquitylated TDP-43 species, such that a reduction in TDP-43 had the positive impact of rescuing UBQLN toxicity [33].

The study in *Drosophila* has revealed an important role of UBQLNs in neurodegenerative diseases in which UBQLNs loss-of-function triggered TDP-43 gain-of-toxic function [33]. Further investigations using these UBQLN’s fly models may contribute to the development of promising novel therapies. For example, we demonstrated in the fly that the genetic manipulation of VCP/p97 activity, which is an important protein in the delivery of large cytoplasmic aggregates to autophagosomes, was sufficient to rescue UBQLN toxicity and the resulting aggregation of TDP-43 [33].

## 3. Perspectives

Neurons are highly susceptible to dysfunction in the ubiquitin-proteasome system, as demonstrated by the wide spectrum of neurodegenerative and neurodevelopmental disorders [153]. Recent findings have shown that oligomer-driven impairments in proteasomal function are relevant to various neurodegenerative diseases, regardless of the specific misfolded proteins involved [154]. Therefore, the stimulation of proteasome degradation has potential as an attractive and promising approach for the development of therapies for proteinopathies including AD, PD, HD and ALS. It is important to note that proteasomes efficiently degrade toxic aggregation-prone proteins as long as they remain in a soluble state [155,156,157].

Increases in the pool of free ubiquitin or overexpressing specific ubiquitin ligases, such as the C-terminus of Hsc-70 interacting protein (CHIP) [158,159,160], has been postulated to enhance UPS activity, which reduces the toxicity induced by protein aggregates. A number of distinct strategies may be taken to enhance proteasome activity, which are briefly summarized in [161]. The overexpression of PA28, one of a series of a positive allosteric regulators of the 20S proteasome, may enhance the survival of neurons in a HD cell-based model [162]. Studies on human fibroblast models have indicated that the up-regulation of proteasome activity is also achieved by the overexpression of proteasome maturation protein (POMP), which increases the levels of functional and assembled proteasomes and enhances the anti-oxidative capacity of cells [163].

Alternatively, the use of small-molecule compounds to stimulate proteasome activity has also been investigated for its therapeutic value in the treatment of neurodegenerative diseases [164,165,166]. For example, the alkaloid canthin-6-one was shown to be effective at accelerating the degradation of α-synuclein by targeting the 26S proteasome non-ATPase regulatory subunit 1 (Rpn2) and activating the proteasome [167]. Enhancements in Tau and Amyloid-β clearance followed by the activation of the proteasome by betulinic acid or rolipram has been demonstrated in MT4 human T-cells and mouse models [168,169]. Some evidence has shown that sulforaphane (SFN) boosts proteasome and autophagy activities to enhance mHTT turnover and cell survival in a cell model of HD [170]. The acceleration of TDP-43 and polyQ protein ataxin-3 proteasomal degradation has been achieved in cell models using IU1, a selective small molecule inhibitor of the deubiquitination enzyme USP14 [166].

Based on the general nature of UPS and its extremely large spectrum of action, an overall increase in degradation by stimulating UPS activity may be accompanied by undesirable side effects. Therefore, the precise and selective targeting of disease-causing proteins for proteasomal degradation is expected to have more advantages than the general stimulation of UPS activity. The pursuit of the biological functions of UbL-UbA family members has highlighted the critical roles of these proteins in the control of proteasome activity and revealed that the genetic manipulation of ubiquitin receptors is an effective regulator of the turnover of some specific neurodegenerative disease-related proteins.

Despite exciting new findings in this field, the functions of the UbL-UbA proteins have not yet been fully characterized and their targets or cross-talk with the proteasome remain unclear. Once the overall targets of each UbL-UbA family protein are fully identified, these proteins may become an attractive molecular knife to use in the onset of neurodegenerative diseases. As such, the functions of UbL-UbA proteins may be improved for selective delivery to proteasomes and the facilitated degradation of their aggregation-prone targets. Alternatively, abnormal and eventually toxic levels of UbL-UbA proteins may be modulated by specific targeting.

The use of *Drosophila* models will provide a novel insight into the possible beneficial use of UbL-UbA proteins as targets in the treatment of neurodegenerative diseases. *Drosophila* is an attractive in vivo model for investigating neurodegeneration for several reasons, including its short life span (~60 days), easy genetic manipulation, rapid screening for mutations, and similar biological complexity to that of mammals. Moreover, critical mechanisms in *Drosophila* neurodegeneration were proven to be regulated in a similar manner in humans. *Drosophila* is also an excellent in vivo model for screening biologically active compounds, as already evidenced by the numerous candidate drug studies that have been undertaken using *Drosophila* models of HD, AD, and PD [171].

As summarized above, *Drosophila* has already been proven as a valid system to examine the involvement of NUB1 in mediating the degradation of mHTT, Rad23 in reducing the SCA3 pathology, and UBQLN2 in inducing the TDP-43 pathology. The further characterization of these models may lead to the identification of promising new therapies for these neurodegenerative diseases.

## Figures and Tables

**Table 1:** Protein identities of *Drosophila* and human UbL-UbA proteins. The primary structures of UbL-UbA members were examined using the Expasy Prosite SIM alignment tool for protein sequences (www.expasy.org/sim) with default parameters.

Name	Uniprot	Human Orthologs	Protein Identity (%)	Domain Identity (%)
**rngo**	Q9VXF9	Ddi1 Ddi2	49.1	UbL (39.7) UbL (39.7)
**NUB1**	Q9VRF3	NUB1	31.9	UbL (30.3) UbA-1 (50) UBA-2 (26.7)
**Rad23/1**	Q9V3W9	Rad23A Rad23A	36.7 36.7	Rad23A/UbL (52.2) Rad23A/UbA-1 (100) Rad23A/UbA-2 (84.2)
**ISOT-3A**	Q86LF0	KPC2	31.3	UbA-1 (37.1) UbA-2 (53.1)
**Ubqn**	Q9VWD9	UBQLN1 UBQLN2 UBQLN4	50.2 47.6 46.5	UBQLN2/UbL (56.3) UBQLN2/UbA (84.6)

## References

[1] Labbadia J., Morimoto R.I. (2015). The Biology of Proteostasis in Aging and Disease. Annu. Rev. Biochem..

[2] Klaips C.L., Jayaraj G.G., Hartl F.U. (2018). Pathways of cellular proteostasis in aging and disease. J. Cell Biol..

[3] Boland B., Yu W.H., Corti O., Mollereau B., Henriques A., Bezard E., Pastores G.M., Rubinsztein D.C., Nixon R.A., Duchen M.R. (2018). Promoting the clearance of neurotoxic proteins in neurodegenerative disorders of ageing. Nat. Rev. Drug Discov..

[4] Grice G.L., Nathan J.A. (2016). The recognition of ubiquitinated proteins by the proteasome. Cell. Mol. Life Sci..

[5] Jang H.H. (2018). Regulation of Protein Degradation by Proteasomes in Cancer. J. Cancer Prev..

[6] Collins G.A., Goldberg A.L. (2017). The Logic of the 26S Proteasome. Cell.

[7] Zhao J., Zhai B., Gygi S.P., Goldberg A.L. (2015). mTOR inhibition activates overall protein degradation by the ubiquitin proteasome system as well as by autophagy. Proc. Natl. Acad. Sci. USA.

[8] Bard J.A.M., Goodall E.A., Greene E.R., Jonsson E., Dong K.C., Martin A. (2018). Structure and Function of the 26S Proteasome. Annu. Rev. Biochem..

[9] Livneh I., Cohen-Kaplan V., Cohen-Rosenzweig C., Avni N., Ciechanover A. (2016). The life cycle of the 26S proteasome: From birth, through regulation and function, and onto its death. Cell Res..

[10] Doherty J., Baehrecke E.H. (2018). Life, death and autophagy. Nat. Cell Biol..

[11] Komander D., Rape M. (2012). The Ubiquitin Code. Annu. Rev. Biochem..

[12] Xu P., Duong D.M., Seyfried N.T., Cheng D., Xie Y., Robert J., Rush J., Hochstrasser M., Finley D., Peng J. (2009). Quantitative Proteomics Reveals the Function of Unconventional Ubiquitin Chains in Proteasomal Degradation. Cell.

[13] Jacobson A.D., Zhang N.-Y., Xu P., Han K.-J., Noone S., Peng J., Liu C.-W. (2009). The Lysine 48 and Lysine 63 Ubiquitin Conjugates Are Processed Differently by the 26 S Proteasome. J. Biol. Chem..

[14] Kisselev A.F., Akopian T.N., Woo K.M., Goldberg A.L. (1999). The sizes of peptides generated from protein by mammalian 26 and 20 S proteasomes. Implications for understanding the degradative mechanism and antigen presentation. J. Biol. Chem..

[15] Palombella V.J., Rando O.J., Goldberg A.L., Maniatis T. (1994). The ubiquitin-proteasome pathway is required for processing the NF-kappa B1 precursor protein and the activation of NF-kappa B. Cell.

[16] Lin L., Ghosh S. (1996). A glycine-rich region in NF-kappaB p105 functions as a processing signal for the generation of the p50 subunit. Mol. Cell. Biol..

[17] Rape M., Jentsch S. (2002). Taking a bite: Proteasomal protein processing. Nat. Cell Biol..

[18] Hoppe T., Matuschewski K., Rape M., Schlenker S., Ulrich H.D., Jentsch S. (2000). Activation of a membrane-bound transcription factor by regulated ubiquitin/proteasome-dependent processing. Cell.

[19] Pan Y., Bai C.B., Joyner A.L., Wang B. (2006). Sonic hedgehog Signaling Regulates Gli2 Transcriptional Activity by Suppressing Its Processing and Degradation. Mol. Cell. Biol..

[20] Yang Y., Zhang J., Ma D., Zhang M., Hu S., Shao S., Gong C.-X. (2013). Subcutaneous administration of liraglutide ameliorates Alzheimer-associated tau hyperphosphorylation in rats with type 2 diabetes. J. Alzheimers Dis..

[21] Kraut D.A. (2013). Slippery substrates impair ATP-dependent protease function by slowing unfolding. J. Biol. Chem..

[22] Keren-Kaplan T., Zeev Peters L., Levin-Kravets O., Attali I., Kleifeld O., Shohat N., Artzi S., Zucker O., Pilzer I., Reis N. (2016). Structure of ubiquitylated-Rpn10 provides insight into its autoregulation mechanism. Nat. Commun..

[23] Lu X., Nowicka U., Sridharan V., Liu F., Randles L., Hymel D., Dyba M., Tarasov S.G., Tarasova N.I., Zhao X.Z. (2017). Structure of the RPN13-RPN2 complex provides insights for Rpn13 and Uch37 as anticancer targets. Nat. Commun..

[24] VanderLinden R.T., Hemmis C.W., Yao T., Robinson H., Hill C.P. (2017). Structure and energetics of pairwise interactions between proteasome subunits RPN2, RPN13, and ubiquitin clarify a substrate recruitment mechanism. J. Biol. Chem..

[25] Mueller T.D., Kamionka M., Feigon J. (2004). Specificity of the Interaction between Ubiquitin-associated Domains and Ubiquitin. J. Biol. Chem..

[26] Kang Y., Vossler R.A., Diaz-Martinez L.A., Winter N.S., Clarke D.J., Walters K.J. (2006). UBL/UBA Ubiquitin Receptor Proteins Bind a Common Tetraubiquitin Chain. J. Mol. Biol..

[27] Husnjak K., Dikic I. (2012). Ubiquitin-Binding Proteins: Decoders of Ubiquitin-Mediated Cellular Functions. Annu. Rev. Biochem..

[28] Buchberger A. (2002). From UBA to UBX: New words in the ubiquitin vocabulary. Trends Cell Biol..

[29] Ciechanover A., Schwartz A.L. (2004). The ubiquitin system: Pathogenesis of human diseases and drug targeting. Biochim. Biophys. Acta Mol. Cell Res..

[30] Hipp M.S., Kasturi P., Hartl F.U. The proteostasis network and its decline in ageing. Nat. Rev. Mol. Cell Biol..

[31] Kurtishi A., Rosen B., Patil K.S., Alves G.W., Møller S.G. (2018). Cellular Proteostasis in Neurodegeneration. Mol. Neurobiol..

[32] Pace M.C., Xu G., Fromholt S., Howard J., Crosby K., Giasson B.I., Lewis J., Borchelt D.R. (2018). Changes in proteome solubility indicate widespread proteostatic disruption in mouse models of neurodegenerative disease. Acta Neuropathol..

[33] Jantrapirom S., Lo Piccolo L., Yoshida H., Yamaguchi M. (2018). Depletion of Ubiquilin induces an augmentation in soluble ubiquitinated *Drosophila* TDP-43 to drive neurotoxicity in the fly. Biochim. Biophys. Acta Mol. Basis Dis..

[34] Le N.T.T., Chang L., Kovlyagina I., Georgiou P., Safren N., Braunstein K.E., Kvarta M.D., Van Dyke A.M., LeGates T.A., Philips T. (2016). Motor neuron disease, TDP-43 pathology, and memory deficits in mice expressing ALS–FTD-linked *UBQLN2* mutations. Proc. Natl. Acad. Sci. USA.

[35] Liu Y., Dai H., Xiao W. (1997). UAS MAG1, a yeast cis-acting element that regulates the expression of MAG1, is located within the protein coding region of DDI1. Mol. Gen. Genet. MGG.

[36] Zhu Y., Xiao W. (2001). Two alternative cell cycle checkpoint pathways differentially control DNA damage-dependent induction of MAG1 and DDI1 expression in yeast. Mol. Genet. Genom..

[37] Jelinsky S.A., Samson L.D. (1999). Global response of Saccharomyces cerevisiae to an alkylating agent. Proc. Natl. Acad. Sci. USA.

[38] Gabriely G., Kama R., Gelin-Licht R., Gerst J.E. (2008). Different domains of the UBL-UBA ubiquitin receptor, Ddi1/Vsm1, are involved in its multiple cellular roles. Mol. Biol. Cell.

[39] Nowicka U., Zhang D., Walker O., Krutauz D., Castañeda C.A., Chaturvedi A., Chen T.Y., Reis N., Glickman M.H., Fushman D. (2015). DNA-damage-inducible 1 protein (Ddi1) contains an uncharacteristic ubiquitin-like domain that binds ubiquitin. Structure.

[40] Bertolaet B.L., Clarke D.J., Wolff M., Watson M.H., Henze M., Divita G., Reed S.I. (2001). UBA domains of DNA damage-inducible proteins interact with ubiquitin. Nat. Struct. Biol..

[41] Trempe J.-F., Brown N.R., Lowe E.D., Gordon C., Campbell I.D., Noble M.E.M., Endicott J.A. (2005). Mechanism of Lys48-linked polyubiquitin chain recognition by the Mud1 UBA domain. EMBO J..

[42] Sivá M., Svoboda M., Veverka V., Trempe J.-F., Hofmann K., Kožíšek M., Hexnerová R., Sedlák F., Belza J., Brynda J. (2016). Human DNA-Damage-Inducible 2 Protein Is Structurally and Functionally Distinct from Its Yeast Ortholog. Sci. Rep..

[43] Díaz-Martínez L.A., Kang Y., Walters K.J., Clarke D.J. (2006). Yeast UBL-UBA proteins have partially redundant functions in cell cycle control. Cell Div..

[44] Sirkis R., Gerst J.E., Fass D. (2006). Ddi1, a Eukaryotic Protein With the Retroviral Protease Fold. J. Mol. Biol..

[45] Rosenzweig R., Bronner V., Zhang D., Fushman D., Glickman M.H. (2012). Rpn1 and Rpn2 coordinate ubiquitin processing factors at proteasome. J. Biol. Chem..

[46] Gomez T.A., Kolawa N., Gee M., Sweredoski M.J., Deshaies R.J. (2011). Identification of a functional docking site in the Rpn1 LRR domain for the UBA-UBL domain protein Ddi1. BMC Biol..

[47] Lehrbach N.J., Ruvkun G. (2016). Proteasome dysfunction triggers activation of SKN-1A/Nrf1 by the aspartic protease DDI-1. Elife.

[48] Arlt A., Bauer I., Schafmayer C., Tepel J., Müerköster S.S., Brosch M., Röder C., Kalthoff H., Hampe J., Moyer M.P. (2009). Increased proteasome subunit protein expression and proteasome activity in colon cancer relate to an enhanced activation of nuclear factor E2-related factor 2 (Nrf2). Oncogene.

[49] Radhakrishnan S.K., Lee C.S., Young P., Beskow A., Chan J.Y., Deshaies R.J. (2010). Transcription factor Nrf1 mediates the proteasome recovery pathway after proteasome inhibition in mammalian cells. Mol. Cell.

[50] Steffen J., Seeger M., Koch A., Krüger E. (2010). Proteasomal Degradation Is Transcriptionally Controlled by TCF11 via an ERAD-Dependent Feedback Loop. Mol. Cell.

[51] Alexander J., Kalev O., Mehrabian S., Traykov L., Raycheva M., Kanakis D., Drineas P., Lutz M.I., Ströbel T., Penz T. (2016). Familial early-onset dementia with complex neuropathologic phenotype and genomic background. Neurobiol. Aging.

[52] Morawe T., Honemann-Capito M., von Stein W., Wodarz A. (2011). Loss of the extraproteasomal ubiquitin receptor Rings lost impairs ring canal growth in *Drosophila* oogenesis. J. Cell Biol..

[53] Ramirez J., Lectez B., Osinalde N., Sivá M., Elu N., Aloria K., Procházková M., Perez C., Martínez-Hernández J., Barrio R. (2018). Quantitative proteomics reveals neuronal ubiquitination of Rngo/Ddi1 and several proteasomal subunits by Ube3a, accounting for the complexity of Angelman syndrome. Hum. Mol. Genet..

[54] Buiting K., Williams C., Horsthemke B. (2016). Angelman syndrome—Insights into a rare neurogenetic disorder. Nat. Rev. Neurol..

[55] Kito K., Yeh E.T.H., Kamitani T. (2001). NUB1, a NEDD8-interacting Protein, Is Induced by Interferon and Down-regulates the NEDD8 Expression. J. Biol. Chem..

[56] Schmidtke G., Kalveram B., Groettrup M. (2009). Degradation of FAT10 by the 26S proteasome is independent of ubiquitylation but relies on NUB1L. FEBS Lett..

[57] Hipp M.S., Raasi S., Groettrup M., Schmidtke G. (2004). NEDD8 ultimate buster-1L interacts with the ubiquitin-like protein FAT10 and accelerates its degradation. J. Biol. Chem..

[58] Tanji K., Tanaka T., Kamitani T. (2005). Interaction of NUB1 with the proteasome subunit S5a. Biochem. Biophys. Res. Commun..

[59] Rani N., Aichem A., Schmidtke G., Kreft S.G., Groettrup M. (2012). FAT10 and NUB1L bind to the VWA domain of Rpn10 and Rpn1 to enable proteasome-mediated proteolysis. Nat. Commun..

[60] Kamitani T., Kito K., Fukuda-Kamitani T., Yeh E.T.H. (2001). Targeting of NEDD8 and Its Conjugates for Proteasomal Degradation by NUB1. J. Biol. Chem..

[61] Bonacci T., Audebert S., Camoin L., Baudelet E., Iovanna J.-L., Soubeyran P. (2017). Regulation of NUB1 Activity through Non-Proteolytic Mdm2-Mediated Ubiquitination. PLoS ONE.

[62] Tanaka T., Kawashima H., Yeh E.T.H., Kamitani T. (2003). Regulation of the NEDD8 Conjugation System by a Splicing Variant, NUB1L. J. Biol. Chem..

[63] Liu S., Yang H., Zhao J., Zhang Y.-H., Song A.-X., Hu H.-Y. (2013). NEDD8 ultimate buster-1 long (NUB1L) protein promotes transfer of NEDD8 to proteasome for degradation through the P97UFD1/NPL4 complex. J. Biol. Chem..

[64] Kondo H., Rabouille C., Newman R., Levine T.P., Pappin D., Freemont P., Warren G. (1997). p47 is a cofactor for p97-mediated membrane fusion. Nature.

[65] Meyer H.H., Shorter J.G., Seemann J., Pappin D., Warren G. (2000). A complex of mammalian Ufd1 and Npl4 links the AAA-ATPase, p97, to ubiquitin and nuclear transport pathways. EMBO J..

[66] Li J., Ma W., Li H., Hou N., Wang X., Kim I., Li F., Su H. (2015). NEDD8 Ultimate Buster 1 Long (NUB1L) Protein Suppresses Atypical Neddylation and Promotes the Proteasomal Degradation of Misfolded Proteins. J. Biol. Chem..

[67] Liu G., Xirodimas D.P. (2010). NUB1 promotes cytoplasmic localization of p53 through cooperation of the NEDD8 and ubiquitin pathways. Oncogene.

[68] Mori F., Tanji K., Odagiri S., Hattori M., Hoshikawa Y., Kono C., Yasui K., Yokoi S., Hasegawa Y., Kamitani T. (2012). Ubiquitin-related proteins in neuronal and glial intranuclear inclusions in intranuclear inclusion body disease. Pathol. Int..

[69] Tanji K., Mori F., Kakita A., Zhang H., Kito K., Kamitani T., Takahashi H., Wakabayashi K. (2007). Immunohistochemical localization of NUB1, a synphilin-1-binding protein, in neurodegenerative disorders. Acta Neuropathol..

[70] Tanji K., Mori F., Kito K., Kakita A., Mimura J., Itoh K., Takahashi H., Kamitani T., Wakabayashi K. (2011). Synphilin-1-Binding Protein NUB1 is Colocalized With Nonfibrillar, Proteinase K-Resistant α-Synuclein in Presynapses in Lewy Body Disease. J. Neuropathol. Exp. Neurol..

[71] Tanji K., Tanaka T., Mori F., Kito K., Takahashi H., Wakabayashi K., Kamitani T. (2006). NUB1 Suppresses the Formation of Lewy Body-Like Inclusions by Proteasomal Degradation of Synphilin-1. Am. J. Pathol..

[72] Hooper C., Killick R., Lovestone S. (2008). The GSK3 hypothesis of Alzheimer’s disease. J. Neurochem..

[73] Lucas J.J., Hernández F., Gómez-Ramos P., Morán M.A., Hen R., Avila J. (2001). Decreased nuclear beta-catenin, tau hyperphosphorylation and neurodegeneration in GSK-3beta conditional transgenic mice. EMBO J..

[74] Johnson G.V.W., Stoothoff W.H. (2004). Tau phosphorylation in neuronal cell function and dysfunction. J. Cell Sci..

[75] Rankin C.A., Sun Q., Gamblin T.C. (2008). Pre-assembled tau filaments phosphorylated by GSK-3b form large tangle-like structures. Neurobiol. Dis..

[76] Richet E., Pooler A.M., Rodriguez T., Novoselov S.S., Schmidtke G., Groettrup M., Hanger D.P., Cheetham M.E., van der Spuy J. (2012). NUB1 modulation of GSK3β reduces tau aggregation. Hum. Mol. Genet..

[77] Lu B., Al-Ramahi I., Valencia A., Wang Q., Berenshteyn F., Yang H., Gallego-Flores T., Ichcho S., Lacoste A., Hild M. (2013). Identification of NUB1 as a suppressor of mutant Huntingtin toxicity via enhanced protein clearance. Nat. Neurosci..

[78] MacDonald M.E., Ambrose C.M., Duyao M.P., Myers R.H., Lin C., Srinidhi L., Barnes G., Taylor S.A., James M., Groot N. (1993). A novel gene containing a trinucleotide repeat that is expanded and unstable on Huntington’s disease chromosomes. The Huntington’s Disease Collaborative Research Group. Cell.

[79] Yokoi M., Hanaoka F. (2017). Two mammalian homologs of yeast Rad23, HR23A and HR23B, as multifunctional proteins. Gene.

[80] Dantuma N.P., Heinen C., Hoogstraten D. (2009). The ubiquitin receptor Rad23: At the crossroads of nucleotide excision repair and proteasomal degradation. DNA Repair.

[81] Hiyama H., Yokoi M., Masutani C., Sugasawa K., Maekawa T., Tanaka K., Hoeijmakers J.H., Hanaoka F. (1999). Interaction of hHR23 with S5a. The ubiquitin-like domain of hHR23 mediates interaction with S5a subunit of 26 S proteasome. J. Biol. Chem..

[82] Chen L., Madura K. (2002). Rad23 promotes the targeting of proteolytic substrates to the proteasome. Mol. Cell. Biol..

[83] Elsasser S., Chandler-Militello D., Müller B., Hanna J., Finley D. (2004). Rad23 and Rpn10 Serve as Alternative Ubiquitin Receptors for the Proteasome. J. Biol. Chem..

[84] Kim I., Mi K., Rao H. (2004). Multiple interactions of rad23 suggest a mechanism for ubiquitylated substrate delivery important in proteolysis. Mol. Biol. Cell.

[85] Elsasser S., Gali R.R., Schwickart M., Larsen C.N., Leggett D.S., Müller B., Feng M.T., Tübing F., Dittmar G.A.G., Finley D. (2002). Proteasome subunit Rpn1 binds ubiquitin-like protein domains. Nat. Cell Biol..

[86] Chen L., Shinde U., Ortolan T.G., Madura K. (2001). Ubiquitin-associated (UBA) domains in Rad23 bind ubiquitin and promote inhibition of multi-ubiquitin chain assembly. EMBO Rep..

[87] Raasi S., Pickart C.M. (2003). Rad23 ubiquitin-associated domains (UBA) inhibit 26 S proteasome-catalyzed proteolysis by sequestering lysine 48-linked polyubiquitin chains. J. Biol. Chem..

[88] Raasi S., Orlov I., Fleming K.G., Pickart C.M. (2004). Binding of polyubiquitin chains to ubiquitin-associated (UBA) domains of HHR23A. J. Mol. Biol..

[89] Kang Y., Zhang N., Koepp D.M., Walters K.J. (2007). Ubiquitin receptor proteins hHR23a and hPLIC2 interact. J. Mol. Biol..

[90] Heinen C., Acs K., Hoogstraten D., Dantuma N.P. (2011). C-terminal UBA domains protect ubiquitin receptors by preventing initiation of protein degradation. Nat. Commun..

[91] Liang R.-Y., Chen L., Ko B.-T., Shen Y.-H., Li Y.-T., Chen B.-R., Lin K.-T., Madura K., Chuang S.-M. (2014). Rad23 Interaction with the Proteasome Is Regulated by Phosphorylation of Its Ubiquitin-Like (UbL) Domain. J. Mol. Biol..

[92] Medicherla B., Kostova Z., Schaefer A., Wolf D.H. (2004). A genomic screen identifies Dsk2p and Rad23p as essential components of ER-associated degradation. EMBO Rep..

[93] Gödderz D., Heinen C., Marchese F.P., Kurz T., Acs K., Dantuma N.P. (2015). Cdc48-independent proteasomal degradation coincides with a reduced need for ubiquitylation. Sci. Rep..

[94] Kim I., Ahn J., Liu C., Tanabe K., Apodaca J., Suzuki T., Rao H. (2006). The Png1–Rad23 complex regulates glycoprotein turnover. J. Cell Biol..

[95] Joshi S., Katiyar S., Lennarz W.J. (2005). Misfolding of glycoproteins is a prerequisite for peptide: *N*-glycanase mediated deglycosylation. FEBS Lett..

[96] Hwang G.-W., Sasaki D., Naganuma A. (2005). Overexpression of Rad23 confers resistance to methylmercury in saccharomyces cerevisiae via inhibition of the degradation of ubiquitinated proteins. Mol. Pharmacol..

[97] Glockzin S., Ogi F.-X., Hengstermann A., Scheffner M., Blattner C. (2003). Involvement of the DNA repair protein hHR23 in p53 degradation. Mol. Cell. Biol..

[98] Brignone C., Bradley K.E., Kisselev A.F., Grossman S.R. (2004). A post-ubiquitination role for MDM2 and hHR23A in the p53 degradation pathway. Oncogene.

[99] Blount J.R., Tsou W.-L., Ristic G., Burr A.A., Ouyang M., Galante H., Scaglione K.M., Todi S.V. (2014). Ubiquitin-binding site 2 of ataxin-3 prevents its proteasomal degradation by interacting with Rad23. Nat. Commun..

[100] Paulson H.L., Shakkottai V.G., Clark H.B., Orr H.T. (2017). Polyglutamine spinocerebellar ataxias—From genes to potential treatments. Nat. Rev. Neurosci..

[101] Lundgren J., Masson P., Mirzaei Z., Young P. (2005). Identification and characterization of a *Drosophila* proteasome regulatory network. Mol. Cell. Biol..

[102] Tsou W.-L., Ouyang M., Hosking R.R., Sutton J.R., Blount J.R., Burr A.A., Todi S.V. (2015). The deubiquitinase ataxin-3 requires Rad23 and DnaJ-1 for its neuroprotective role in *Drosophila melanogaster*. Neurobiol. Dis..

[103] Sutton J.R., Blount J.R., Libohova K., Tsou W.-L., Joshi G.S., Paulson H.L., Costa M.d.C., Scaglione K.M., Todi S.V. (2017). Interaction of the polyglutamine protein ataxin-3 with Rad23 regulates toxicity in *Drosophila* models of Spinocerebellar Ataxia Type 3. Hum. Mol. Genet..

[104] Kamura T., Hara T., Matsumoto M., Ishida N., Okumura F., Hatakeyama S., Yoshida M., Nakayama K., Nakayama K.I. (2004). Cytoplasmic ubiquitin ligase KPC regulates proteolysis of p27 Kip1 at G1 phase. Nat. Cell Biol..

[105] Kotoshiba S., Kamura T., Hara T., Ishida N., Nakayama K.I. (2005). Molecular Dissection of the Interaction between p27 and Kip1 Ubiquitylation-promoting Complex, the Ubiquitin Ligase That Regulates Proteolysis of p27 in G_1_ Phase. J. Biol. Chem..

[106] Hara T., Kamura T., Kotoshiba S., Takahashi H., Fujiwara K., Onoyama I., Shirakawa M., Mizushima N., Nakayama K.I. (2005). Role of the UBL-UBA Protein KPC2 in Degradation of p27 at G1 Phase of the Cell Cycle. Mol. Cell. Biol..

[107] Bridoux L., Bergiers I., Draime A., Halbout M., Deneyer N., Twizere J.-C., Rezsohazy R. (2015). KPC2 relocalizes HOXA2 to the cytoplasm and decreases its transcriptional activity. Biochim. Biophys. Acta Gene Regul. Mech..

[108] Mah A.L., Perry G., Smith M.A., Monteiro M.J. (2000). Identification of ubiquilin, a novel presenilin interactor that increases presenilin protein accumulation. J. Cell Biol..

[109] Conklin D., Holderman S., Whitmore T.E., Maurer M., Feldhaus A.L. (2000). Molecular cloning, chromosome mapping and characterization of UBQLN3 a testis-specific gene that contains an ubiquitin-like domain. Gene.

[110] Marín I. (2014). The ubiquilin gene family: Evolutionary patterns and functional insights. BMC Evol. Biol..

[111] Deng H.-X., Chen W., Hong S.-T., Boycott K.M., Gorrie G.H., Siddique N., Yang Y., Fecto F., Shi Y., Zhai H. (2011). Mutations in UBQLN2 cause dominant X-linked juvenile and adult-onset ALS and ALS/dementia. Nature.

[112] Teyssou E., Chartier L., Amador M.-D.-M., Lam R., Lautrette G., Nicol M., Machat S., Da Barroca S., Moigneu C., Mairey M. (2017). Novel UBQLN2 mutations linked to amyotrophic lateral sclerosis and atypical hereditary spastic paraplegia phenotype through defective HSP70-mediated proteolysis. Neurobiol. Aging.

[113] Sharkey L.M., Safren N., Pithadia A.S., Gerson J.E., Dulchavsky M., Fischer S., Patel R., Lantis G., Ashraf N., Kim J.H. (2018). Mutant UBQLN2 promotes toxicity by modulating intrinsic self-assembly. Proc. Natl. Acad. Sci. USA.

[114] Hjerpe R., Bett J.S., Keuss M.J., Solovyova A., McWilliams T.G., Johnson C., Sahu I., Varghese J., Wood N., Wightman M. (2016). UBQLN2 Mediates Autophagy-Independent Protein Aggregate Clearance by the Proteasome. Cell.

[115] Kleijnen M.F., Alarcon R.M., Howley P.M. (2003). The ubiquitin-associated domain of hPLIC-2 interacts with the proteasome. Mol. Biol. Cell.

[116] Seok Ko H., Uehara T., Tsuruma K., Nomura Y. (2004). Ubiquilin interacts with ubiquitylated proteins and proteasome through its ubiquitin-associated and ubiquitin-like domains. FEBS Lett..

[117] Walters K.J., Kleijnen M.F., Goh A.M., Wagner G., Howley P.M. (2002). Structural studies of the interaction between ubiquitin family proteins and proteasome subunit S5a. Biochemistry.

[118] Xia Y., Yan L.H., Huang B., Liu M., Liu X., Huang C. (2014). Pathogenic mutation of UBQLN2 impairs its interaction with UBXD8 and disrupts endoplasmic reticulum-associated protein degradation. J. Neurochem..

[119] Lim P.J., Danner R., Liang J., Doong H., Harman C., Srinivasan D., Rothenberg C., Wang H., Ye Y., Fang S. (2009). Ubiquilin and p97/VCP bind erasin, forming a complex involved in ERAD. J. Cell Biol..

[120] Kim T.-Y., Kim E., Yoon S.K., Yoon J.-B. (2008). Herp enhances ER-associated protein degradation by recruiting ubiquilins. Biochem. Biophys. Res. Commun..

[121] Xie Z., Klionsky D.J. (2007). Autophagosome formation: Core machinery and adaptations. Nat. Cell Biol..

[122] N’Diaye E.N., Kajihara K.K., Hsieh I., Morisaki H., Debnath J., Brown E.J. (2009). PLIC proteins or ubiquilins regulate autophagy-dependent cell survival during nutrient starvation. EMBO Rep..

[123] Huang S., Li Y., Yuan X., Zhao M., Wang J., Li Y., Li Y., Lin H., Zhang Q., Wang W. (2019). The UbL-UBA Ubiquilin4 protein functions as a tumor suppressor in gastric cancer by p53-dependent and p53-independent regulation of p21. Cell Death Differ..

[124] Kessler R., Tisserand J., Font-Burgada J., Reina O., Coch L., Attolini C.S., Garcia-Bassets I., Azorín F. (2015). dDsk2 regulates H2Bub1 and RNA polymerase II pausing at dHP1c complex target genes. Nat. Commun..

[125] Hartmann-Petersen R., Wallace M., Hofmann K., Koch G., Johnsen A.H., Hendil K.B., Gordon C. (2004). The Ubx2 and Ubx3 cofactors direct Cdc48 activity to proteolytic and nonproteolytic ubiquitin-dependent processes. Curr. Biol..

[126] Regan-Klapisz E., Sorokina I., Voortman J., de Keizer P., Roovers R.C., Verheesen P., Urbé S., Fallon L., Fon E.A., Verkleij A. (2005). Ubiquilin recruits Eps15 into ubiquitin-rich cytoplasmic aggregates via a UIM-UBL interaction. J. Cell Sci..

[127] Kleijnen M.F., Shih A.H., Zhou P., Kumar S., Soccio R.E., Kedersha N.L., Gill G., Howley P.M. (2000). The hPLIC proteins may provide a link between the ubiquitination machinery and the proteasome. Mol. Cell.

[128] Massey L.K., Mah A.L., Ford D.L., Miller J., Liang J., Doong H., Monteiro M.J. (2004). Overexpression of ubiquilin decreases ubiquitination and degradation of presenilin proteins. J. Alzheimers Dis..

[129] Bedford F.K., Kittler J.T., Muller E., Thomas P., Uren J.M., Merlo D., Wisden W., Triller A., Smart T.G., Moss S.J. (2001). GABAA receptor cell surface number and subunit stability are regulated by the ubiquitin-like protein Plic-1. Nat. Neurosci..

[130] Zhang D., Raasi S., Fushman D. (2008). Affinity Makes the Difference: Nonselective Interaction of the UBA Domain of Ubiquilin-1 with Monomeric Ubiquitin and Polyubiquitin Chains. J. Mol. Biol..

[131] Kaye F.J., Modi S., Ivanovska I., Koonin E.V., Thress K., Kubo A., Kornbluth S., Rose M.D. (2000). A family of ubiquitin-like proteins binds the ATPase domain of Hsp70-like Stch. FEBS Lett..

[132] Haapasalo A., Viswanathan J., Bertram L., Soininen H., Tanzi R.E., Hiltunen M. (2010). Emerging role of Alzheimer’s disease-associated ubiquilin-1 in protein aggregation: Figure 1. Biochem. Soc. Trans..

[133] El Ayadi A., Stieren E.S., Barral J.M., Boehning D. (2013). Ubiquilin-1 and protein quality control in Alzheimer disease. Prion.

[134] Zhang K.Y., Yang S., Warraich S.T., Blair I.P. (2014). Ubiquilin 2: A component of the ubiquitin-proteasome system with an emerging role in neurodegeneration. Int. J. Biochem. Cell Biol..

[135] Ling S.-C., Polymenidou M., Cleveland D.W. (2013). Converging Mechanisms in ALS and FTD: Disrupted RNA and Protein Homeostasis. Neuron.

[136] Viswanathan J., Haapasalo A., Böttcher C., Miettinen R., Kurkinen K.M.A., Lu A., Thomas A., Maynard C.J., Romano D., Hyman B.T. (2011). Alzheimer’s Disease-Associated Ubiquilin-1 Regulates Presenilin-1 Accumulation and Aggresome Formation. Traffic.

[137] Stieren E.S., El Ayadi A., Xiao Y., Siller E., Landsverk M.L., Oberhauser A.F., Barral J.M., Boehning D. (2011). Ubiquilin-1 is a molecular chaperone for the amyloid precursor protein. J. Biol. Chem..

[138] Daoud H., Suhail H., Szuto A., Camu W., Salachas F., Meininger V., Bouchard J.-P., Dupré N., Dion P.A., Rouleau G.A. (2012). UBQLN2 mutations are rare in French and French–Canadian amyotrophic lateral sclerosis. Neurobiol. Aging.

[139] Synofzik M., Maetzler W., Grehl T., Prudlo J., vom Hagen J.M., Haack T., Rebassoo P., Munz M., Schöls L., Biskup S. (2012). Screening in ALS and FTD patients reveals 3 novel UBQLN2 mutations outside the PXX domain and a pure FTD phenotype. Neurobiol. Aging.

[140] Williams K.L., Warraich S.T., Yang S., Solski J.A., Fernando R., Rouleau G.A., Nicholson G.A., Blair I.P. (2012). UBQLN2/ubiquilin 2 mutation and pathology in familial amyotrophic lateral sclerosis. Neurobiol. Aging.

[141] Brettschneider J., Van Deerlin V.M., Robinson J.L., Kwong L., Lee E.B., Ali Y.O., Safren N., Monteiro M.J., Toledo J.B., Elman L. (2012). Pattern of ubiquilin pathology in ALS and FTLD indicates presence of C9ORF72 hexanucleotide expansion. Acta Neuropathol..

[142] Riley B.E., Xu Y., Zoghbi H.Y., Orr H.T. (2004). The effects of the polyglutamine repeat protein ataxin-1 on the UbL-UBA protein A1Up. J. Biol. Chem..

[143] Yang H., Yue H.-W., He W.-T., Hong J.-Y., Jiang L.-L., Hu H.-Y. (2018). PolyQ-expanded huntingtin and ataxin-3 sequester ubiquitin adaptors hHR23B and UBQLN2 into aggregates via conjugated ubiquitin. FASEB J..

[144] Rutherford N.J., Lewis J., Clippinger A.K., Thomas M.A., Adamson J., Cruz P.E., Cannon A., Xu G., Golde T.E., Shaw G. (2013). Unbiased screen reveals ubiquilin-1 and -2 highly associated with huntingtin inclusions. Brain Res..

[145] Kim S.H., Stiles S.G., Feichtmeier J.M., Ramesh N., Zhan L., Scalf M.A., Smith L.M., Bhan Pandey U., Tibbetts R.S. (2018). Mutation-dependent aggregation and toxicity in a Drosophila model for UBQLN2-associated ALS. Hum. Mol. Genet..

[146] Hanson K.A., Kim S.H., Wassarman D.A., Tibbetts R.S. (2010). Ubiquilin Modifies TDP-43 Toxicity in a Drosophila Model of Amyotrophic Lateral Sclerosis (ALS). J. Biol. Chem..

[147] Kim S.H., Shi Y., Hanson K.A., Williams L.M., Sakasai R., Bowler M.J., Tibbetts R.S. (2009). Potentiation of Amyotrophic Lateral Sclerosis (ALS)-associated TDP-43 Aggregation by the Proteasome-targeting Factor, Ubiquilin 1. J. Biol. Chem..

[148] Picher-Martel V., Dutta K., Phaneuf D., Sobue G., Julien J.P. (2015). Ubiquilin-2 drives NF-κB activity and cytosolic TDP-43 aggregation in neuronal cells. Mol. Brain.

[149] Gkazi S.A., Troakes C., Topp S., Miller J.W., Vance C.A., Sreedharan J., Al-Chalabi A., Kirby J., Shaw P.J., Al-Sarraj S. (2019). Striking phenotypic variation in a family with the P506S UBQLN2 mutation including amyotrophic lateral sclerosis, spastic paraplegia, and frontotemporal dementia. Neurobiol. Aging.

[150] Osaka M., Ito D., Suzuki N. (2016). Disturbance of proteasomal and autophagic protein degradation pathways by amyotrophic lateral sclerosis-linked mutations in ubiquilin 2. Biochem. Biophys. Res. Commun..

[151] Li A., Xie Z., Dong Y., McKay K.M., McKee M.L., Tanzi R.E. (2007). Isolation and characterization of the *Drosophila* ubiquilin ortholog dUbqln: In vivo interaction with early-onset Alzheimer disease genes. Hum. Mol. Genet..

[152] Jantrapirom S., Lo Piccolo L., Yoshida H., Yamaguchi M. (2017). A new *Drosophila* model of Ubiquilin knockdown shows the effect of impaired proteostasis on locomotive and learning abilities. Exp. Cell Res..

[153] Atkin G., Paulson H. (2014). Ubiquitin pathways in neurodegenerative disease. Front. Mol. Neurosci..

[154] Thibaudeau T.A., Anderson R.T., Smith D.M. (2018). A common mechanism of proteasome impairment by neurodegenerative disease-associated oligomers. Nat. Commun..

[155] Venkatraman P., Wetzel R., Tanaka M., Nukina N., Goldberg A.L. (2004). Eukaryotic proteasomes cannot digest polyglutamine sequences and release them during degradation of polyglutamine-containing proteins. Mol. Cell.

[156] Holmberg C.I., Staniszewski K.E., Mensah K.N., Matouschek A., Morimoto R.I. (2004). Inefficient degradation of truncated polyglutamine proteins by the proteasome. EMBO J..

[157] Jana N.R., Zemskov E.A., Wang G.H., Nukina N. (2001). Altered proteasomal function due to the expression of polyglutamine-expanded truncated N-terminal huntingtin induces apoptosis by caspase activation through mitochondrial cytochrome c release. Hum. Mol. Genet..

[158] Lee F.K.M., Wong A.K.Y., Lee Y.W., Wan O.W., Edwin Chan H.Y., Chung K.K.K. (2009). The role of ubiquitin linkages on α-synuclein induced-toxicity in a *Drosophila* model of Parkinson’s disease. J. Neurochem..

[159] Al-Ramahi I., Lam Y.C., Chen H.-K., de Gouyon B., Zhang M., Pérez A.M., Branco J., de Haro M., Patterson C., Zoghbi H.Y. (2006). CHIP Protects from the Neurotoxicity of Expanded and Wild-type Ataxin-1 and Promotes Their Ubiquitination and Degradation. J. Biol. Chem..

[160] Tsai Y.C., Fishman P.S., Thakor N.V., Oyler G.A. (2003). Parkin Facilitates the Elimination of Expanded Polyglutamine Proteins and Leads to Preservation of Proteasome Function. J. Biol. Chem..

[161] Schmidt M., Finley D. (2014). Regulation of proteasome activity in health and disease. Biochim. Biophys. Acta Mol. Cell Res..

[162] Seo H., Sonntag K.-C., Kim W., Cattaneo E., Isacson O. (2007). Proteasome Activator Enhances Survival of Huntington’s Disease Neuronal Model Cells. PLoS ONE.

[163] Chondrogianni N., Gonos E.S. (2007). Overexpression of hUMP1/POMP proteasome accessory protein enhances proteasome-mediated antioxidant defence. Exp. Gerontol..

[164] Leestemaker Y., de Jong A., Witting K.F., Penning R., Schuurman K., Rodenko B., Zaal E.A., van de Kooij B., Laufer S., Heck A.J.R. (2017). Proteasome Activation by Small Molecules. Cell Chem. Biol..

[165] Dantuma N.P., Bott L.C. (2014). The ubiquitin-proteasome system in neurodegenerative diseases: Precipitating factor, yet part of the solution. Front. Mol. Neurosci..

[166] Lee B.-H., Lee M.J., Park S., Oh D.-C., Elsasser S., Chen P.-C., Gartner C., Dimova N., Hanna J., Gygi S.P. (2010). Enhancement of proteasome activity by a small-molecule inhibitor of USP14. Nature.

[167] Yuan N.-N., Cai C.-Z., Wu M.-Y., Zhu Q., Su H., Li M., Ren J., Tan J.-Q., Lu J.-H. (2019). Canthin-6-One Accelerates Alpha-Synuclein Degradation by Enhancing UPS Activity: Drug Target Identification by CRISPR-Cas9 Whole Genome-Wide Screening Technology. Front. Pharmacol..

[168] Huang L., Ho P., Chen C.-H. (2007). Activation and inhibition of the proteasome by betulinic acid and its derivatives. FEBS Lett..

[169] Myeku N., Clelland C.L., Emrani S., Kukushkin N.V., Yu W.H., Goldberg A.L., Duff K.E. (2016). Tau-driven 26S proteasome impairment and cognitive dysfunction can be prevented early in disease by activating cAMP-PKA signaling. Nat. Med..

[170] Liu Y., Hettinger C.L., Zhang D., Rezvani K., Wang X., Wang H. (2014). Sulforaphane enhances proteasomal and autophagic activities in mice and is a potential therapeutic reagent for Huntington’s disease. J. Neurochem..

[171] Newman T., Sinadinos C., Johnston A., Sealey M., Mudher A. (2011). Using *Drosophila* models of neurodegenerative diseases for drug discovery. Expert Opin. Drug Discov..

